# G3BP1 inhibits RNA virus replication by positively regulating RIG-I-mediated cellular antiviral response

**DOI:** 10.1038/s41419-019-2178-9

**Published:** 2019-12-11

**Authors:** Wenping Yang, Yi Ru, Jingjing Ren, Juncui Bai, Junshu Wei, Shaozu Fu, Xiangtao Liu, Dan Li, Haixue Zheng

**Affiliations:** State Key Laboratory of Veterinary Etiological Biology and OIE/National Foot and Mouth Disease Reference Laboratory, Lanzhou Veterinary Research Institute, Chinese Academy of Agricultural Sciences, Lanzhou, 730046 Gansu China

**Keywords:** Extracellular signalling molecules, RIG-I-like receptors

## Abstract

Retinoic acid-inducible gene I (RIG-I) is a pattern recognition receptor and is involved in the innate immune response against RNA viruses infection. Here, we demonstrate that the Ras-GTPase-activating protein SH3-domain-binding protein 1 (G3BP1) serves as a positive regulator of the RIG-I-mediated signaling pathway. G3BP1-deficient cells inhibited RNA virus-triggered induction of downstream antiviral genes. Furthermore, we found that G3BP1 inhibited the replication of Sendai virus and vesicular stomatitis virus, indicating a positive regulation of G3BP1 to cellular antiviral responses. Mechanistically, G3BP1 formed a complex with RNF125 and RIG-I, leading to decreased RNF125 via its auto-ubiquitination; thus, promoting expression of RIG-I. Overall, the results suggest a novel mechanism for G3BP1 in the positive regulation of antiviral signaling mediated by RIG-I.

## Introduction

The innate immune system defenses against invading microbial pathogens via the recognition of pathogen-associated molecular patterns (PAMPs) by a range of pattern recognition receptors (PRRs)^1,2^. The PRR family is divided into four different classes, such as transmembrane proteins [Toll-like receptors (TLRs) and C-type lectin receptors (CLRs)] and cytoplasmic proteins [retinoic acid-inducible gene (RIG)-I-like receptors (RLRs) and NOD-like receptors (NLRs)]^1,3,4^. It is widely accepted that the RLR family is composed of RIG-I, melanoma differentiation-associated gene 5 (MDA5), and laboratory of genetics and physiology 2 (LGP2)^5–8^.

RIG-I activity is regulated by a series of post-translational modifications, such as poly-ubiquitination, sumoylation, and phosphorylation^9–14^. In particular, ubiquitination is crucial for regulating RIG-I activity. The E3 ubiquitin ligases TRIM25 and Riplet (also known as RNF135) have been reported to catalyze the K63-linked poly-ubiquitination of RIG-I, thereby leading to a significant increase in RIG-I downstream signaling activities^9,15,16^. In contrast, RIG-I undergoes degradation after conjugation to E3 ubiquitin ligases RNF125 and RNF122^13,17^. Moreover, many host proteins are involved in the regulation of RIG-I-mediated innate immune signaling. For instance, cyclophilin A (CypA) positively regulates the antiviral response by increasing TRIM25-mediated K63-linked ubiquitination of RIG-I^18^, which is also boosted by the interaction between 14-3-3ε and RIG-I^19^. DDX6 acts as an RNA co-sensor through colocalization with RIG-I, resulting in the augmentation of innate immune signaling^20^.

Ras-GTPase activating SH3-domain-binding-protein 1 (G3BP1), a multi-domain protein, has been identified to be evolutionarily conserved from yeast to human^21^. G3BP1 is considered as a critical component of mammalian cell stress granules (SG), which are formed when cells are undergoing stress and where host mRNAs are sequestered and translationally arrested^22,23^. A recent study has demonstrated that G3BP1 is pivotal for the efficient activation of cGAS and for DNA sensing by promoting the formation of large cGAS complexes^24^. In addition, G3BP1 binds to viral dsRNA and RIG-I to enhance IFN-β response^25^. However, the effect of G3BP1 on RNA virus replication and the molecular mechanism remains unclear.

In the current study, our data suggest that G3BP1 inhibits the replication of Sendai virus (SeV) and vesicular stomatitis virus (VSV). Mechanistically, G3BP1 suppresses RNF125-mediated K48-linked ubiquitination of RIG-I to increase RIG-I expression and positively regulates antiviral innate immunity.

## Materials and methods

### Reagents, virus, and cells

Antibodies against Myc-tag, G3BP1, RIG-I, p-P65, P65, p-IRF3, IRF3, p-IκBα, and IκBα were obtained from Cell Signaling Technology; anti-Flag, anti-HA, and anti-β-actin were from Sigma; horseradish peroxidase (HRP)-conjugated anti-mouse IgG and anti-rabbit IgG were from Thermo; HRP-conjugated anti-goat IgG was from Zhong Shan Jin Qiao; MG132 and 3-MA were from Sigma; RNF125 and K48-Ub were from Abcam; Ub was from Santa Cruz Biotechnology; 5´ppp-dsRNA was from InvivoGen; EZ-link Psoralen-PEG3-Biotin and Streptavidin agarose resin were from Thermo Fisher. SeV and VSV-GFP were described previously^26,27^. The cultivation of human embryonic kidney (HEK) 293 T cells have been described previously^28^.

### Constructs

Luciferase reporter plasmids under the control of Nifty, ISRE, the IFN-β promoter and mammalian expression plasmids encoding RIG-I (CARD), MDA5, MAVS (also known as VISA), TBK1, IKKε, TRAF6, IRF3, and IRF7 were generated as described previously^29,30^. CMV promoter-based mammalian expression plasmids encoding HA-, Myc-, or Flag-tagged G3BP1 and HA- or Flag-tagged RNF125 were constructed via standard molecular biology techniques. Mammalian expression plasmids for G3BP1 or RNF125 mutants were constructed by a standard mutagenesis method.

### Reporter assays

HEK293T cells (1 × 10^5^) were transfected with the corresponding plasmids by standard calcium phosphate precipitation for 12 h. Meanwhile, 20 ng of pRL-TK (Renilla luciferase) reporter plasmid was added to each transfection to normalize the transfection efficiency. Then adual-specific luciferase assay kit (Promega) was used for performing the luciferase reporter assays. Firefly luciferase activity was measured and normalized to Renilla luciferase activity. The experiment was repeated in triplicate.

### Co-immunoprecipitation (Co-IP) and western blot analysis

For transient transfection and Co-IP experiments, HEK293T cells (2 × 10^6^) were transfected with the indicated plasmids for 24 h. Cells were lysed with 1 ml of lysis buffer (15 mM Tris, 150 mM NaCl, 1% Triton, 25 mM KCl, 2 mM EGTA, 2 mM EDTA, 0.1 mM dithiothreitol, 0.5% Triton X-100, 10 μg/ml aprotinin, 10 μg/ml leupeptin, and 0.5 mM phenylmethylsulfonyl fluoride, pH 7.5). Then a 0.4-ml aliquot of lysate was incubated with 0.2 μg of the indicated monoclonal antibody or control mouse IgG and 20 μl of protein G agarose beads (Amersham Biosciences) at 4 °C for 2 h. The beads were washed three times with 1 ml of lysis buffer containing 0.5 M NaCl and the precipitates were analyzed using western blot. For endogenous immunoprecipitation experiments, cells were uninfected or infected with 0.1 MOI of SeV for the indicated time. Cells were then harvested and lysed in 5 ml of lysis buffer, and the lysate was incubated with 1 μg of the indicated antiserum or pre-immune control serum. The subsequent procedures were conducted as described before. Each western blot assay was repeated at least three times.

### Quantitative real-time polymerase chain reaction (qRT-PCR)

Total RNA was isolated from HEK293T cells utilizing TRIzol reagent (Invitrogen) according to the manufacturer’s instruction. qRT-PCR analysis was carried out to measure transcription of *IFNB1*, *RANTES*, *TNFa*, *IL-8*, *IL-6*, *IP-10*, *ISG56*, SeV *P*, VSV *P*, and *GAPDH* genes, with the following primers: *IFNB1*, 5′-cagcaattttcagtgtcagaagct-3′ and 5′-cagtgactgtactccttggcctt-3′; *RANTENS*, 5′-ggcagccctcgctgtcatcc-3′ and 5′-gcagcagggtgtggtgtccg-3′; *TNFa*, 5′-gccgcatcgccgtctcctac-3′ and 5′-cctcagccccctctggggtc-3′; *IL-8*, 5′-gagagtgattgagagtggaccac-3′ and 5′-cacaaccctctgcacccagttt-3′; *IL-6*, 5′-ttctccacaagcgccttcggtc-3′ and 5′-tctgtgtggggcggctacatct-3′; *IP-10*, 5′-ggtgagaagagatgtctgaatcc-3′ and 5′-gtccatccttggaagcactgca-3′; *ISG56*, 5′-acggctgcctaatttacagc-3′ and 5′-agtggctgatatctgggtgc-3′; SeV *P*: 5′-caaaagtgagggcgaaggagaa-3′ and 5′- cgcccagatcctgagatacaga-3′; VSV *P*: 5′-gtgacggacgaatgtctcataa-3′ and 5′-tttgactctcgcctgattgtac-3′; *GAPDH*, 5′-aaaatcaagtggggcgatgct-3′; and 5′-gggcagagatgatgacccttt-3′. GAPDH was applied to normalize the relative abundance of indicated mRNA.

### RNA interference

Double-strand oligonucleotides corresponding to the target sequences were cloned into the pSuper.retro RNAi plasmid (Oligoengine). The target sequences for human G3BP1 cDNA were as follows: #1: 5′-ggattggattcaaatggaaag-3′; #2: 5′-ggagattcatgcaaacgtttg-3′. A pSuper.retro RNAi plasmid targeting GFP was used as a control for all RNAi-related experiments.

### In vivo ubiquitination assays

Cells were lysed in 100 μl lysis buffer, and the supernatant proteins were denatured at 95 °C for 5 min in the presence of 1% SDS. Then the denatured lysates were diluted with lysis buffer until the concentration of SDS was reduced to 0.1%, followed by immunoprecipitation (denatured-IP) was performed with the indicated antibodies. The immunoprecipitants were then subjected to immunoblotting with anti-ubiquitin or anti-K48-linked ubiquitin chains.

### In vitro pull-down assays

5´ppp-dsRNA was conjugated with biotin by UV (365 nm wave-length) cross-linking for 45 min. HEK293T cells transfected with the indicated plasmids were lysed with NP-40 lysis buffer. Lysates were incubated with biotinylated-5´ppp-dsRNA at 4 °C for 1 h, and then incubated with streptavidin beads at 4 °C for another 2 h. The beads were washed three times with lysis buffer and the proteins were analyzed by immunoblotting.

### Transfection

A control-RNAi or G3BP1-RNAi retroviral plasmid (10 μg) was co-transfected into HEK293T cells (1 × 10^6^) with two packaging plasmids pGag-Pol (10 μg) and pVSV-G (3 μg) by calcium phosphate precipitation. Cells were washed 12 h post-transfection and replenished with antibiotic-free medium for additional 24 h. The recombinant virus-containing medium was filtered and used to infect HEK293T cells in the presence of polybrene (4 μg/ml, Millipore). The infected HEK293T cells were then selected with puromycin (0.5 μg/ml) for 14 days prior to further experiments.

### Generation of G3BP1-knockout cell lines by CRISPR-Cas9

To generate G3BP1-knockout cell lines, double-stranded oligonucleotides (G3BP1: 5′-tacttggtctgggtcccctt-3′) corresponding to the target sequences

were cloned into the lenti-CRISPR-V2 vector and co-transfected into HEK293T cells with packaging plasmids for 24 h. HEK293T cells were infected with lentivirus for 24 h, followed by selection with puromycin (1 μg/ml, Amresco) for 14 days.

### Statistical analysis

Statistical tests were performed using Microsoft Excel. The significance of differences between samples was assessed using an unpaired two-tailed Student *t*-est. The variance was estimated by calculating the standard deviation (SD) and is represented by error bars. Experiments were repeated as described in the figure legends, with a representative experiment being shown. Statistical significance was denoted as follows: *P < 0.05; **P < 0.01.

## Results

### G3BP1 overexpression potentiates RNA virus-triggered signaling

**Fig. 1:**
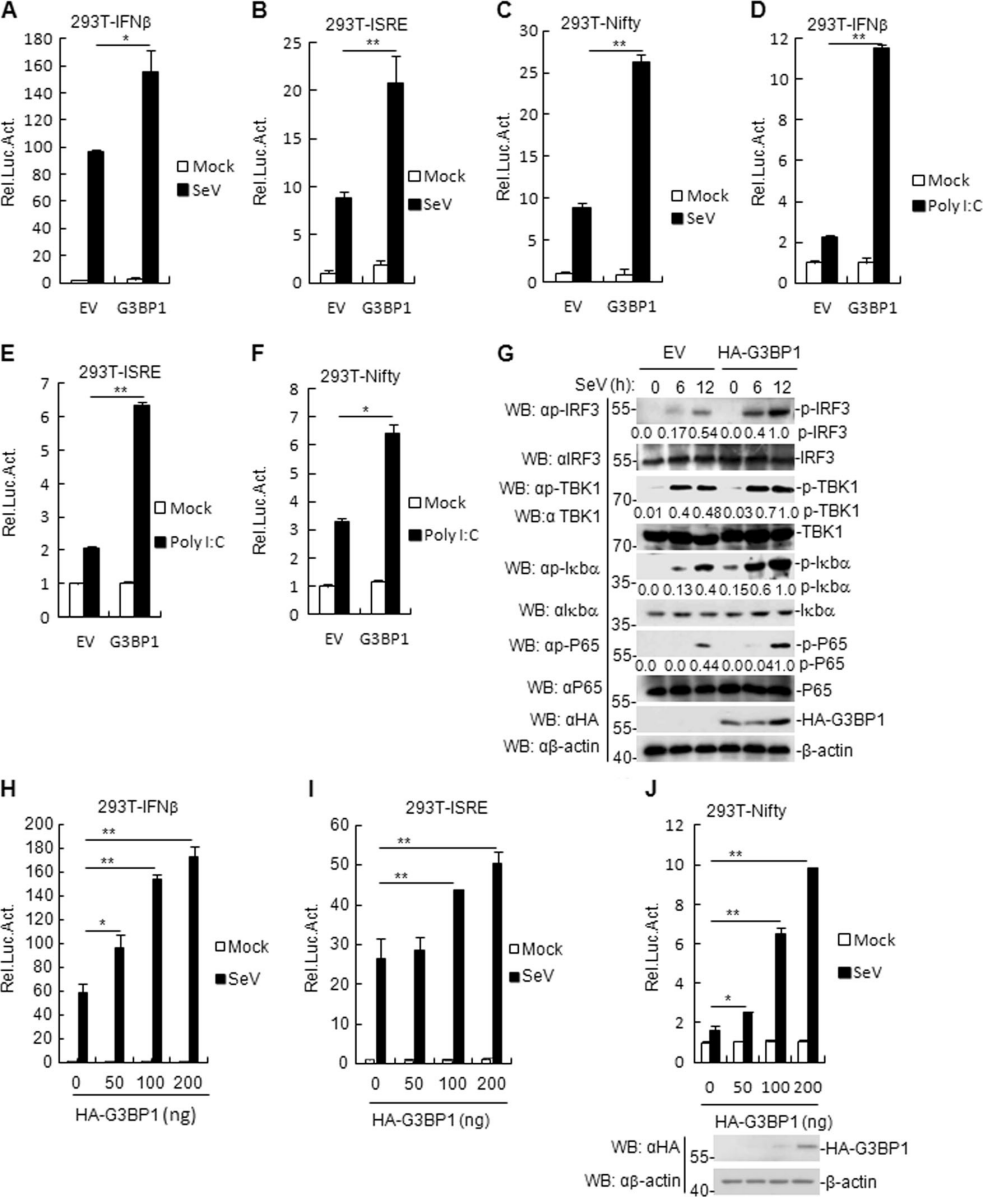
G3BP1 positively regulates SeV-triggered signaling. **a**–**c** G3BP1 activates SeV-induced IFN-β promoter, ISRE, and Nifty. HEK293T cells (1 × 10^5^) were transfected with IFN-β reporter, ISRE, and Nifty (0.1 μg), TK (0.02 μg), and G3BP1 plasmids (0.1 μg) for 24 h and then uninfected or infected with SeV for 12 h before luciferase assays were performed. The experiment was repeated in triplicates. **d**–**f** G3BP1 activates poly (I:C)-induced IFN-β promoter, ISRE, and Nifty. HEK293T cells (1 × 10^5^) were transfected with IFN-β reporter, ISRE and Nifty (0.1 μg), TK (0.02 μg) and G3BP1 plasmids (0.1 μg) for 24 h and then transfected with poly (I:C) (1 μg/ml) for 18 h before luciferase assays were performed. The experiment was repeated in triplicates. **g** Effects of G3BP1 overexpression on SeV-induced phosphorylation of TBK1, IRF3, P65, and Iκbα. HEK293T cells stably overexpressing G3BP1 were infected or uninfected with SeV for the indicated time and then immunoblotting analysis was performed. For the phosphorylation of TBK1, IRF3, P65, and Iκbα, band intensities were determined by Image J software. **h**–**j** G3BP1 activates the IFN-β promoter, ISRE, and Nifty in a dose-dependent manner. HEK293T cells (1 × 10^5^) were transfected with the IFN-β reporter (0.1 μg) and increasing amounts of G3BP1 plasmid (0, 50, 100, and 200 ng) for 24 h and then uninfected or infected with SeV for 12 h before luciferase assays were performed. The experiment was repeated in triplicates. Data are mean ± SD of three independent experiments. *P < 0.05, **P < 0.01, two-tailed *t*-test. EV empty vector, Luc luciferase.

Although G3BP1 acts as a SG resident protein, the mechanism mediated by G3BP1 in host innate immunity against RNA viruses is unclear. To assess the role of G3BP1 in the innate immune response to RNA viruses, we first constructed a flag-tagged G3BP1 expression plasmid and performed reporter assays in HEK293T cells. The results showed that G3BP1 overexpression facilitated both SeV- and RNA analog poly (I:C)-triggered activation of the IFN-β promoter, interferon-stimulated response element (ISRE), and Nifty (Fig. 1a–f) in a dose-dependent manner (Fig. 1h–j). In addition, phosphorylation of TBK1, IRF3, P65, and Iκbα following SeV infection (a hallmark of virus-triggered signaling) was enhanced in G3BP1-overexpressed cells, compared with that in control cells transfected with empty vector (EV) (Fig. 1g). Consistently, qRT-PCR analysis indicated that overexpression of G3BP1 increased the mRNA levels of IFNB1, *IP-10*, *TNFα*, *IL8*, *RANTES*, ISG56, and IL6 genes induced by SeV or poly (I:C) (Supplementary Fig. 1a–n). These results suggest that G3BP1 positively regulates RNA virus-triggered induction of the antiviral response.

### Both G3BP1 knockdown and knockout inhibits SeV- and poly (I:C)-triggered signaling

**Fig. 2:**
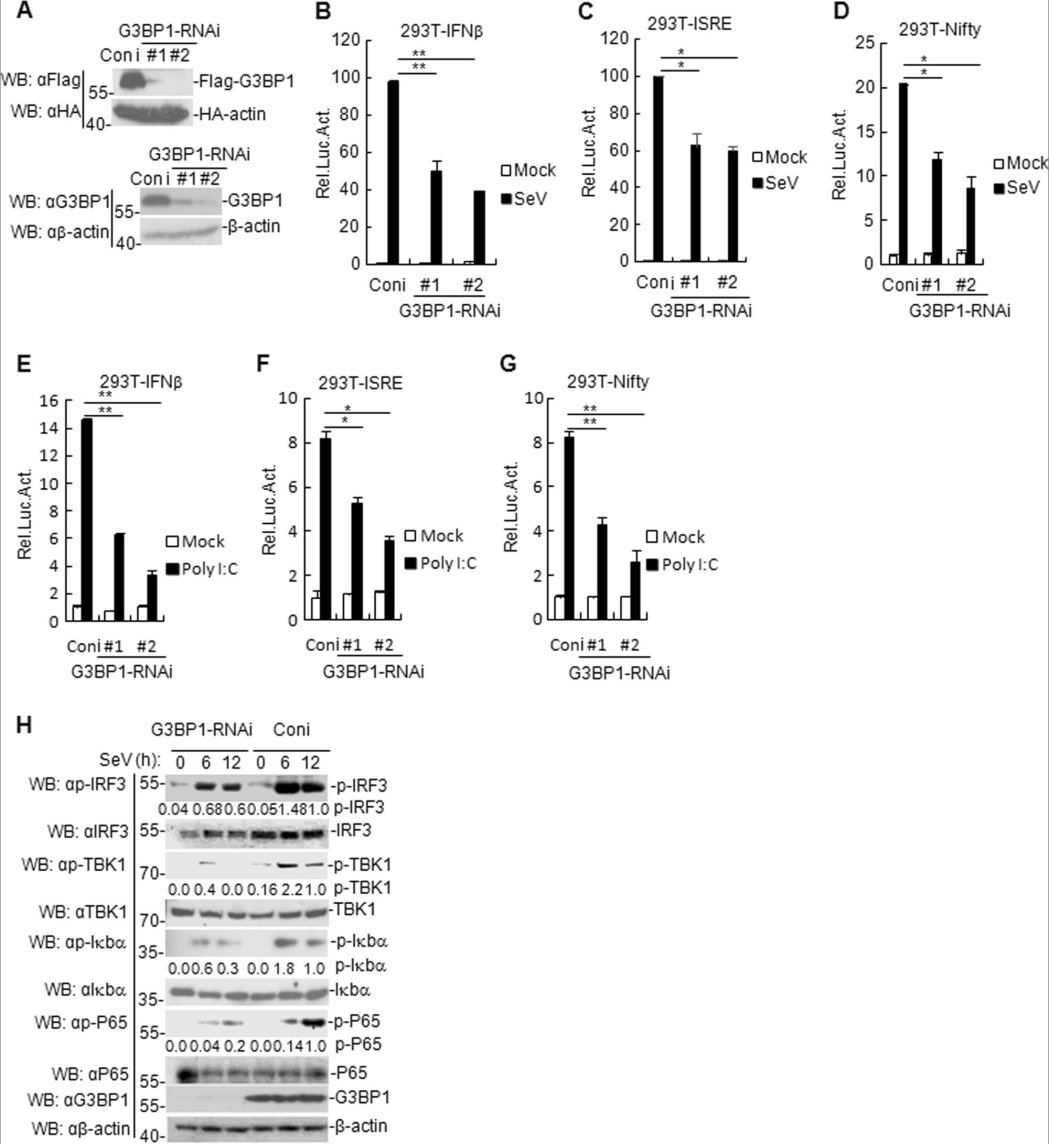
Effects of G3BP1 knockdown on virus-triggered signaling. **a** Effects of G3BP1-RNAi plasmids on exogenous or endogenous G3BP1 expression. **b**–**d** G3BP1 knockdown inhibits SeV-induced IFN-β promoter, ISRE, and Nifty. Stable G3BP1-RNAi knockdown cells (1 × 10^5^) were transfected with the IFN-β reporter, ISRE and Nifty (0.1 μg), and TK (0.02 μg) for 24 h, and then uninfected or infected with SeV for 12 h before luciferase assays. The experiment was repeated in triplicates. **e**–**g** G3BP1 knockdown inhibits poly (I:C)-induced IFN-β promoter, ISRE, and Nifty. Stable G3BP1-knockdown HEK293T cells (1 × 10^5^) were transfected with the IFN-β reporter, ISRE, Nifty (0.1 μg), and TK (0.02 μg) for 24 h, and then transfected with poly (I:C) (1 μg/ml) for 18 h before luciferase assays were performed. The experiment was repeated in triplicates. **h** Effects of G3BP1 knockdown on SeV-induced phosphorylation of TBK1, IRF3, P65, and Iκbα. Stable G3BP1-knockdown HEK293T cells were infected or uninfected with SeV for the indicated time before immunoblotting was performed. For the phosphorylation of TBK1, IRF3, P65, and Iκbα, band intensities were determined by Image J software. Data are mean ± SD of three independent experiments. *P < 0.05, **P < 0.01, two-tailed *t*-test. Coni control-RNAi, Luc luciferase.

To explore whether endogenous G3BP1 is involved in the positive regulation of RIG-I signaling, we first constructed two human G3BP1-RNAi plasmids that efficiently suppressed the expression of both transfected and endogenous G3BP1 in HEK293T cells (Fig. 2a). We found that knockdown of G3BP1 inhibited SeV- and poly (I:C)-triggered activation of the IFN-β promoter, ISRE, and Nifty (Fig. 2b–g). Additionally, SeV- and poly (I:C)-induced phosphorylation of TBK1, IRF3, P65, and Iκbα was diminished in G3BP1-knockdown cells compared with control cells (Fig. 2h). Furthermore, SeV- and poly (I:C)-induced production of IFNB1, *IP-10*, *TNFα*, *IL8*, *RANTES*, ISG56, and IL6 were also attenuated in G3BP1-knockdown cells compared with control cells (Supplementary Fig. 2a–n). Taken together, these results indicate that G3BP1 knockdown negatively regulates RNA virus-triggered antiviral responses.

**Fig. 3:**
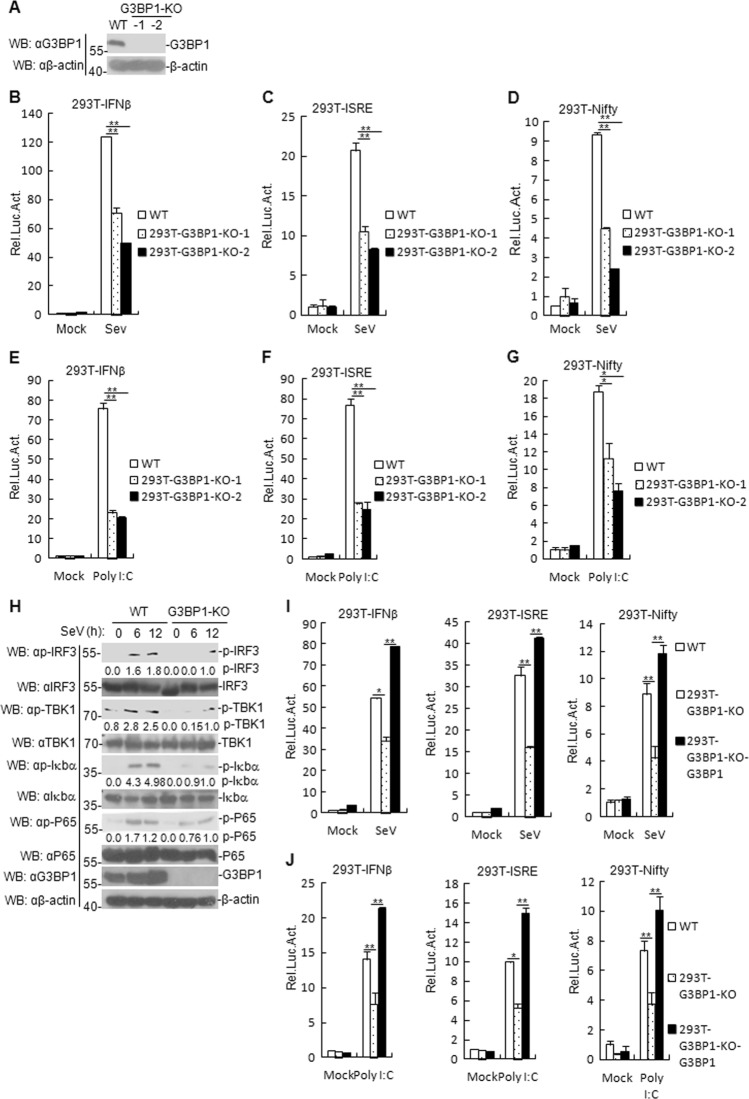
G3BP1-knockout suppresses SeV- and poly (I:C)-triggered signaling. **a** Deficiency of G3BP1 in the KO clones was confirmed by immunoblotting with anti-G3BP1. The G3BP1-deficient HEK293T clones were generated by the CRISPR-Cas9 method. **b**–**g** G3BP1 KO inhibits SeV- or poly (I:C)-induced IFN-β promoter, ISRE, and Nifty. G3BP1-deficient HEK293T cells (1 × 10^5^) were transfected with the IFN-β reporter, ISRE, and Nifty (0.1 μg), and TK (0.02 μg) for 24 h, and then stimulated with SeV for 12 h or with poly (I:C) (1 μg/ml) for 18 h before luciferase assays were performed. Meanwhile, the unstimulated cells were used as the controls. The experiment was repeated in triplicates. **h** Effects of G3BP1 deficiency on SeV-induced phosphorylation of TBK1, IRF3, P65, and Iκbα. G3BP1-deficient HEK293T cells were uninfected or infected with SeV for the indicated time before immunoblotting was performed. For the phosphorylation of TBK1, IRF3, P65, and Iκbα, band intensities were determined by Image J software. **i**, **j** G3BP1-deficient HEK293T cells were reconstituted with G3BP1 by retroviral-mediated gene transfer. The experiments were similarly described in **b**. Data are mean ± SD of three independent experiments. *P < 0.05, **P < 0.01, two-tailed *t*-test. KO knockout, WT wild-type, Luc luciferase.

We next examined if G3BP1-knockout also suppresses SeV- and poly (I:C)-triggered signaling. G3BP1 knockout in mice is lethal at the embryonic stage^31^; therefore, we constructed a G3BP1-deficient HEK293T cell line via CRISPR/Cas9 technology. Immunoblotting analysis result showed that G3BP1 was undetectable in G3BP1-deficient HEK293T cells (Fig. 3a). G3BP1-knockout experiments produced similar results as G3BP1-knockdown experiments (Fig. 3b–h). In addition, reconstitution of G3BP1 in the first G3BP1-deficient cell restored activation of IFN-β promoter, ISRE, and Nifty induced by SeV- and poly (I:C) (Fig. 3i–j). Furthermore, the transcription levels of *IFNB1*, *IP-10*, *TNFα*, *IL8*, *RANTES*, *ISG56*, and *IL6* (Supplementary Fig. 3a–n) in G3BP1-deficient cells were lower than those in wild-type (WT) cells, whereas reconstitution of G3BP1 into the first G3BP1-deficient clone cell restored SeV- or poly (I:C)-induced transcription of the aforementioned downstream genes (Supplementary Fig. 4a–n). Collectively, these data suggest that G3BP1 is essential for the efficient induction of antiviral responses against RNA viruses and cytoplasmic poly (I:C).

### G3BP1 potentiates cellular antiviral responses

**Fig. 4:**
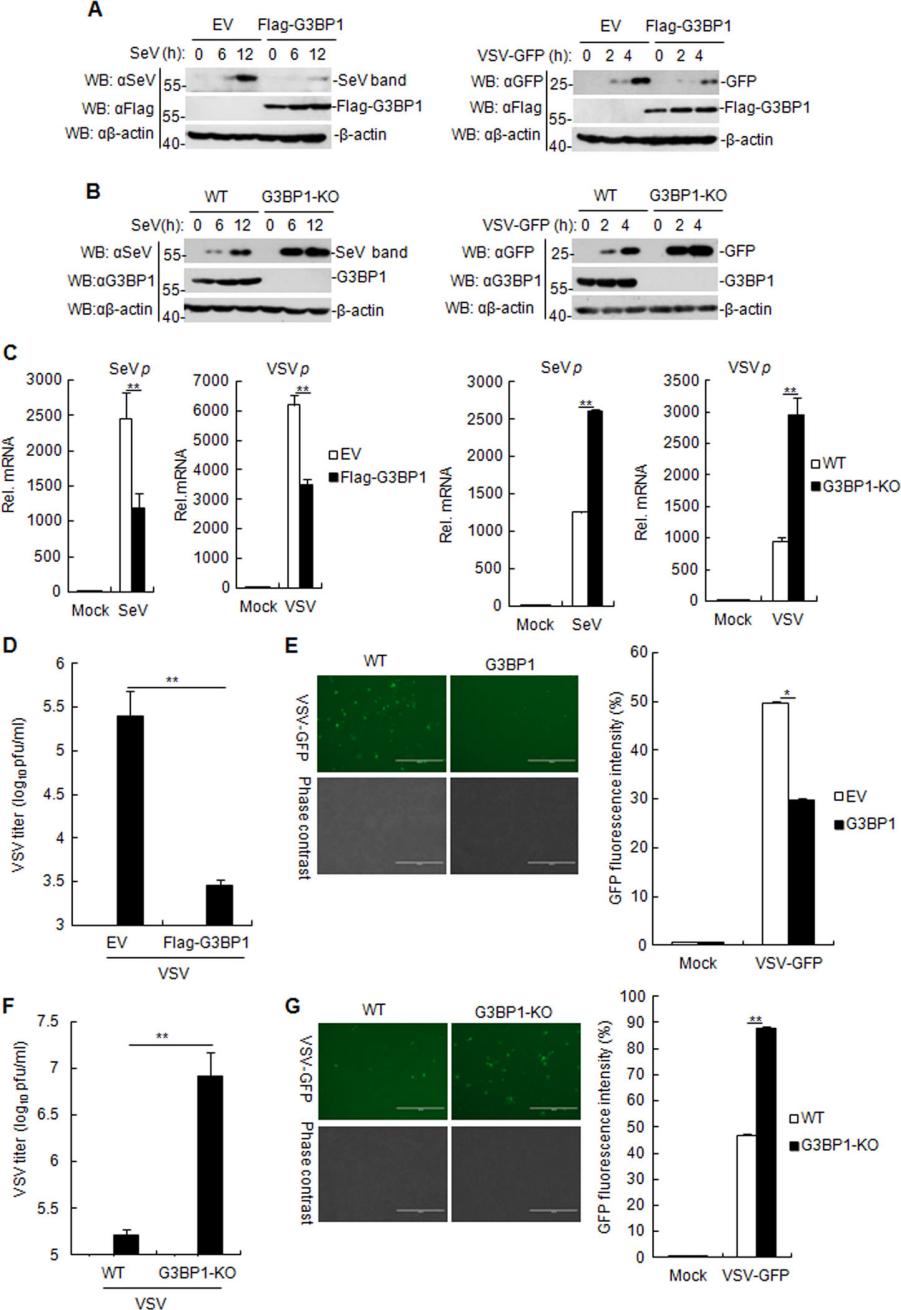
G3BP1 positively regulates the cellular antiviral response. **a**, **b** G3BP1-overexpressed HEK293T cell lines **a** or G3BP1-deficient HEK293T cells **b** were infected with SeV or VSV-GFP (MOI = 0.1) for the indicated time, and then the cell lysates were analyzed by immunoblotting with the antibodies against SeV, GFP, or β-actin. **c** Effects of G3BP1 on SeV and VSV infection. G3BP1-overexpressed or G3BP1-deficient and control HEK293T cells were infected with SeV for 12 h or with VSV-GFP (MOI = 0.1) for 4 h. The mRNA level of the SeV P and VSV P proteins in cells was determined by qRT-PCR. The experiment was repeated in triplicates. **d** Effects of G3BP1-overexpressed on VSV titer. G3BP1-overexpressed HEK293T cells were transfected with 1 μg/ml poly (I:C) for 16 h and infected with VSV-GFP (MOI = 0.1) for 18 h. Supernatants were then analyzed for VSV production by standard plaque assays. The experiment was repeated in triplicates. **e** G3BP1-overexpressed HEK293T cells were infected with VSV-GFP (MOI = 0.1) for 4 h. Images were captured by fluorescence microscopy. In addition, the GFP fluorescence levels in VSV-GFP-infected cells were analyzed by flow cytometry. The experiment was repeated in triplicates. **f** Effects of G3BP1-deficient on VSV titer. The experiments were similarly to those described in **c**. The experiment was repeated in triplicates. **g** G3BP1-deficient HEK293T cells were infected with VSV-GFP (MOI = 0.1) for 2 h. Images were then captured by fluorescence microscopy. In addition, the GFP fluorescence levels in VSV-GFP-infected cells were analyzed by flow cytometry. The experiment was repeated in triplicates. qRT-PCR, quantitative real-time polymerase chain reaction. The experiments were similarly described in **b**. Data are mean ± SD of three independent experiments. *P < 0.05, **P < 0.01, two-tailed *t*-test.

Considering that G3BP1 positively regulates RLR-mediated induction of type I IFNs, we next examined whether G3BP1 affected cellular antiviral responses. The replication of SeV and VSV was evaluated by immunoblotting analysis, using antibodies against viral proteins. As shown in Fig. 4a, the expressions of SeV and GFP proteins in G3BP1-overexpressing cells were lower than those in control cells. In contrast, the replication of both SeV and VSV increased in G3BP1-deficient cells compared with wild-type cells at all examined time points post-infection (Fig. 4b). Accordingly, G3BP1 overexpression inhibited the mRNA level of SeV P and VSV P proteins, whereas G3BP1 knockout exhibited the opposite effect (Fig. 4c). To further confirm these results, VSV replication was measured by immunofluorescence microscopy of VSV tagged with GFP and plaque assays. The results showed that G3BP1 overexpression resulted in decreased VSV replication, as indicated by the lower virus titers (Fig. 4d) and the reduced green fluorescence (Fig. 4e) in the G3BP1-overexpressed cells, suggesting that G3BP1 plays a pivotal role in robust antiviral response. On the contrary, we observed that G3BP1 knockout led to the increased replication of VSV (Fig. 4f–g) in the G3BP1-deficient HEK293T cells. Collectively, these observations suggest that G3BP1 positively regulates cellular antiviral responses.

### G3BP1 targets at upstream of MAVS

**Fig. 5:**
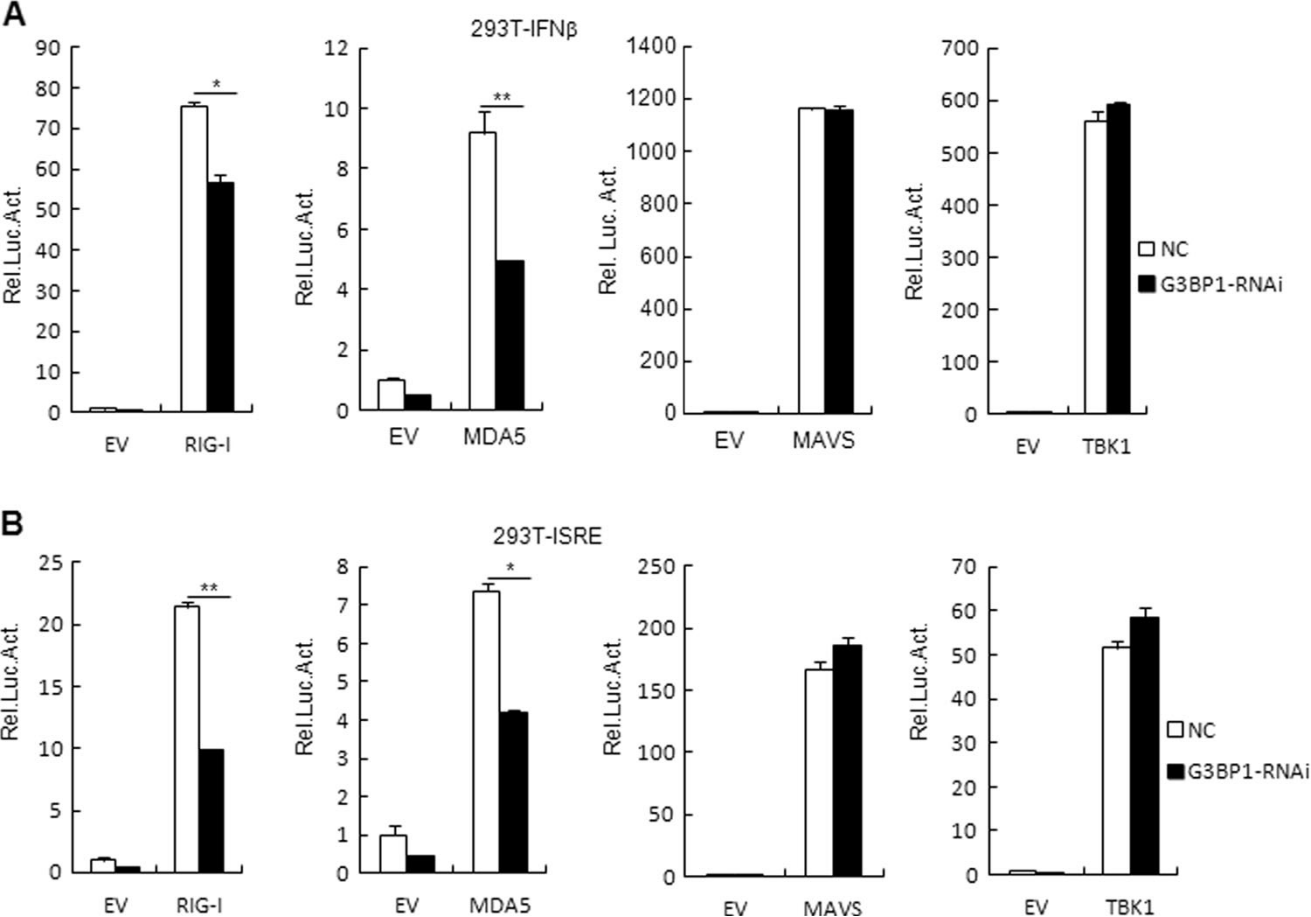
G3BP1 targets at upstream of MAVS. **a**, **b** Effects of G3BP1 knockdown on the IFN-β promoter and ISRE activation. The control or stable G3BP1-knockdown HEK293T cells (1 × 10^5^) were transfected with the IFN-β promoter **a** or ISRE reporter **b** (0.1 μg), TK (0.02 μg), and the indicated plasmids (0.1 μg each) for 24 h followed by luciferase assays. The experiment was repeated in triplicates. Data are mean ± SD of three independent experiments. *P < 0.05, **P < 0.01, two-tailed *t*-test. EV empty vector, Luc luciferase, NC control RNAi.

To identify the potential regulatory targets of G3BP1, we next examined the effects of G3BP1 on the activation of the IFN-β promoter and ISRE mediated by various components of the RLR signaling pathways. Reporter assay results showed that the activation of IFN-β promoter and ISRE mediated by RIG-I and MDA5, but not mediated by MAVS or TBK1, diminished in G3BP1-knockdown cells compared with that of control cells (Fig. 5a–b). These results demonstrate that G3BP1 targets at upstream of MAVS.

### G3BP1 interacts with RIG-I

**Fig. 6:**
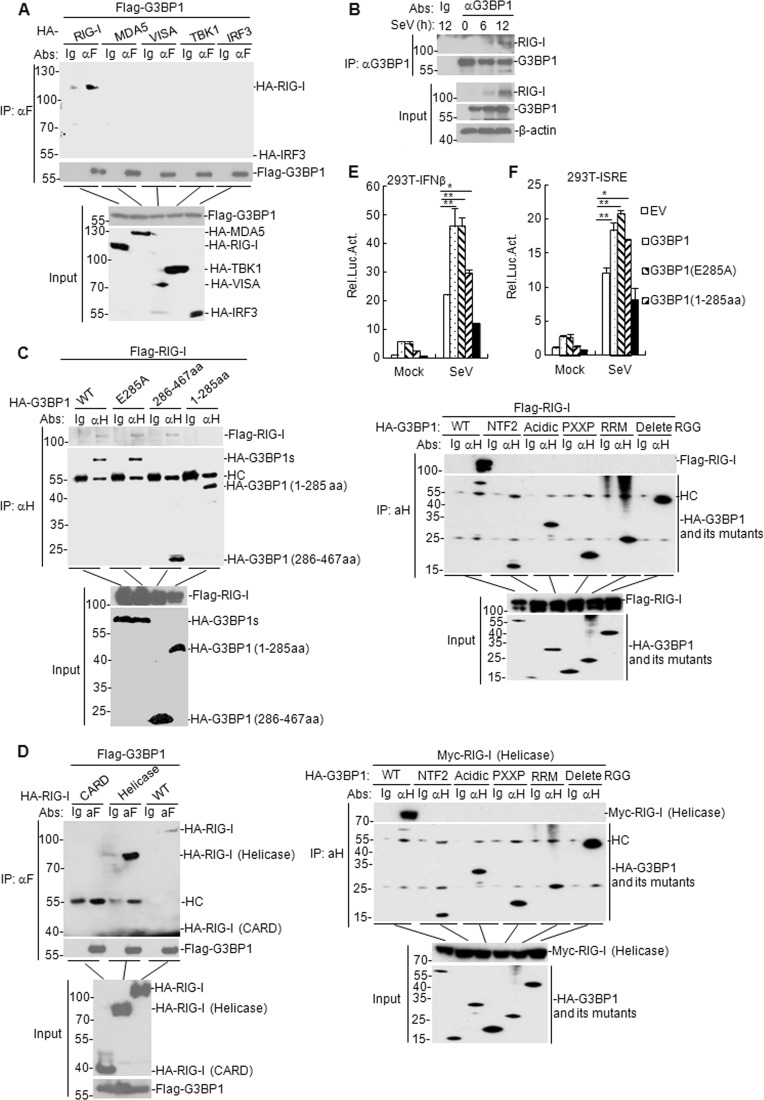
G3BP1 interacts with RIG-I. **a** G3BP1 interacts with RIG-I but not with MDA5, MAVS, TBK1, or IRF3 in the overexpression system. HEK293T cells (2 × 10^6^) were transfected with the indicated plasmids (5 μg of each) for 24 h. Then Co-IP and immunoblotting analysis were performed with the indicated antibodies. **b** Endogenous G3BP1 interacted with RIG-I in HEK293T cells. HEK293T cells (5 × 10^7^) were uninfected or infected with SeV for the indicated time. Co-IP and immunoblotting experiments were performed with the indicated antibodies. **c**, **d** Domain identification of G3BP1 and RIG-I interaction. HEK293T cells were transfected with the expression plasmids encoding RIG-I and G3BP1 or the corresponding mutants (5 μg each) for 24 h. Co-IP and immunoblotting were performed with the indicated antibodies. **e**, **f** Effects of G3BP1 overexpression and its mutants on SeV-triggered IFN-β promoter and ISRE activation. HEK293T cells (1 × 10^5^) were transfected with the IFN-β promoter or ISRE reporter (0.1 μg) and the indicated expression plasmids (0.1 μg) for 24 h. Then cells were uninfected or infected with SeV for 12 h before luciferase assays. The experiment was repeated in triplicates. Data are mean ± SD of three independent experiments. Co-IP Co-immunoprecipitation, EV empty vector, Luc luciferase, αF anti-Flag, αH anti-HA, HC heavy chain, WT wild-type.

We next explored the molecular mechanisms involving G3BP1 in mounting an innate immune responses against RNA viruses. The Co-IP results showed that G3BP1 was associated with RIG-I but not with MDA5, TBK1, MAVS, or IRF3 (Fig. 6a). Furthermore, we found that SeV infection resulted in the gradual interaction between G3BP1 and RIG-I (Fig. 6b). These results suggest that G3BP1 was able to interact with RIG-I.

To identify the domain of G3BP1 mediates its binding to RIG-I, we constructed a series of truncated plasmids for G3BP1 and RIG-I. The glutamic acid-284 (E284) site has been proven to play a role in porcine G3BP1 function^32^. Sequence analysis has indicated that the porcine G3BP1 (E284) site corresponds to the human G3BP1 E285 site; hence, we constructed a human G3BP1 (E285) mutant to investigate its function in innate immunity. We observed that the 286–467 aa domain of G3BP1 and helicase domains of RIG-I were required for the interaction between G3BP1 and RIG-I, while G3BP1 (E285A) mutant did not affect their interaction (Fig. 6c–d). G3BP1 contains five conserved domains: nuclear trans-porter factor 2 (NTF2) domain, acidic domain, PXXP domain, RNA-recognition module (RRM), and RGG (arginine–glycine–glycine) motif^33^. We further found that G3BP1 but not G3BP1 NTF2, G3BP1 acidic domain, G3BP1 PXXP, G3BP1 RRM, and G3BP1 (delete RGG) interacted with RIG-I or RIG-I (helicase) (Fig. 6c–d). In addition, reporter assay results showed that G3BP1 (286–467 aa) mutant, but not G3BP1 (1–285 aa) mutant, participated in RIG-I-mediated activation of IFN-β promoter (Fig. 6e–f). These results indicate that G3BP1 (286–467 aa) domain interacts with RIG-I (helicase) domain and this interaction is essential for the function of G3BP1 in RIG-I-mediated signaling.

### G3BP1 antagonizes RNF125-mediated degradation of RIG-I and enhances the binding of RIG-I to 5´ppp-dsRNA in vivo

**Fig. 7:**
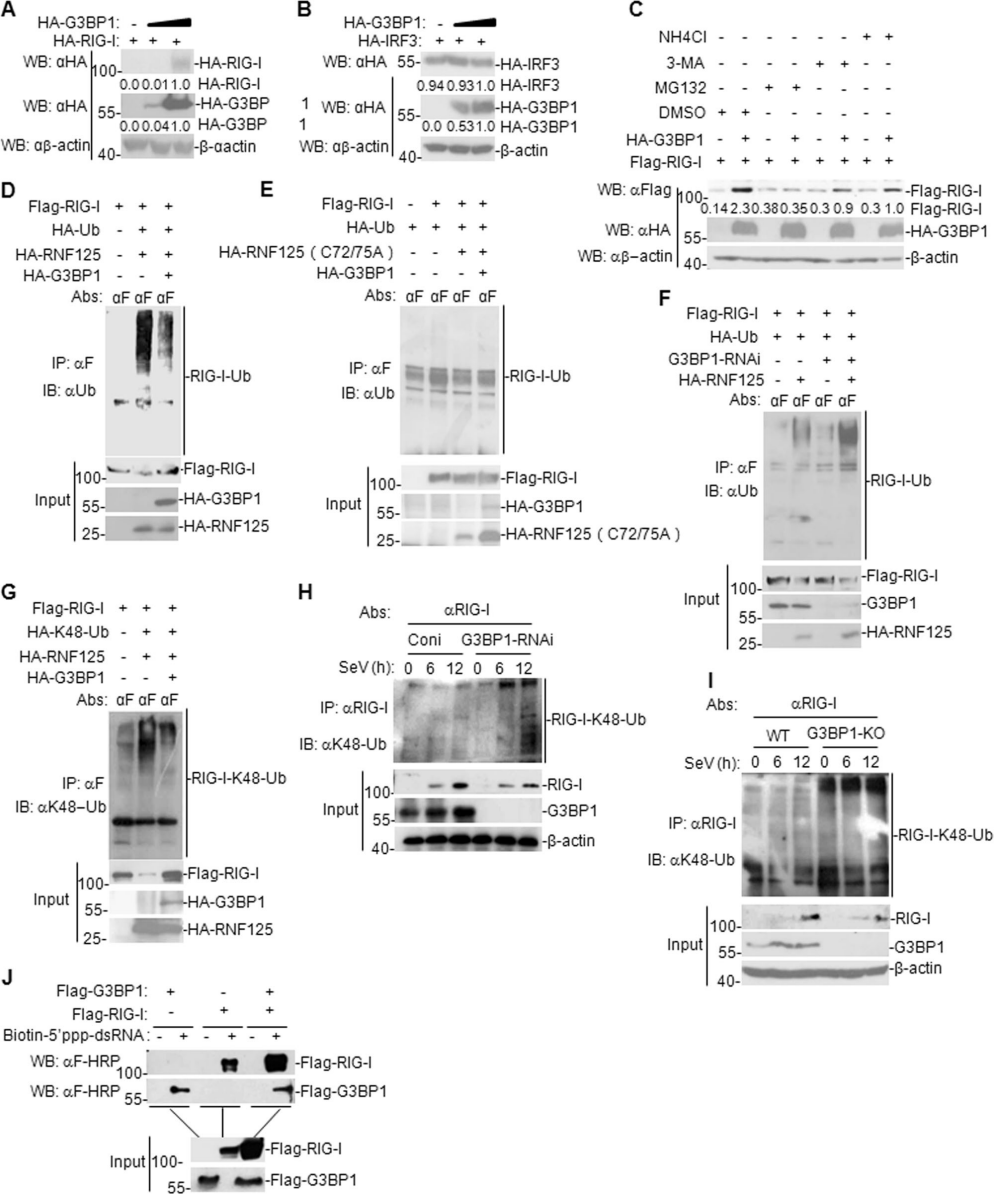
G3BP1 antagonizes RNF125-mediated degradation of RIG-I. **a**, **b** Dose-dependent effects of G3BP1 on the expression of RIG-I or IRF3. HEK293T cells (4 × 10^5^) were transfected with the G3BP1 (0, 1, and 2 μg) and the RIG-I or IRF3 (2 μg) plasmids for 24 h. Then the cell lysates were subjected to immunoblotting with the indicated antibodies. For the HA-RIG-I, HA-G3BP1 and HA-IRF3, band intensities were determined by Image J software. **c** Effects of inhibitors on G3BP1-mediated stabilization of RIG-I. HEK293T cells (4 × 10^5^) were transfected with the indicated plasmids for 18 h and then cells were treated with the indicated inhibitors for 6 h before immunoblotting analysis. For the Flag-RIG-I, band intensities were determined by Image J software. **d**, **e** Effects of G3BP1 on ubiquitination of RIG-I mediated by RNF125 or RNF125 (C72/75 A) mutant. HEK293T cells (2 × 10^6^) were transfected with RIG-I (10 μg), G3BP1 (3 μg), HA-Ub (2 μg), and RNF125 or RNF125 (C72/75 A) (5 μg) for 24 h. Co-IP and immunoblotting analysis were performed with the indicated antibodies. **f** Effects of G3BP1 knockdown on RNF125-mediated ubiquitination of RIG-I. The stable G3BP1-knockdown HEK293T cells were transfected with RIG-I (10 μg), HA-Ub (2 μg), and RNF125 (5 μg) for 24 h. Then Co-IP and immunoblotting were performed with the indicated antibodies. **g** Effects of G3BP1 on K48-linked ubiquitination of RIG-I mediated by RNF125. The experiments were similarly to those described in **d**. **h** Effects of G3BP1 knockdown on K48-linked ubiquitination of RIG-I. The stable G3BP1-knockdown HEK293T cells were infected or uninfected with SeV for the indicated time. Then Co-IP and immunoblotting analysis were performed with the indicated antibodies. **i** Effects of G3BP1 knockout on K48-linked ubiquitination of RIG-I. The experiments were similarly to those described in **h**. **j** G3BP1 binds to 5´ppp-dsRNA and enhances the binding of RIG-I to 5´ppp-dsRNA. HEK293T cells (2 × 10^6^) were transfected with the indicated plasmids (5 μg each). Cell lysates were first incubated with biotinylated-5´ppp-dsRNA and streptavidin-Sepharose. Then conjugated proteins were analyzed by immunoblotting with anti-Flag and anti-HA antibodies. Co-IP Co-immunoprecipitation, αF anti-Flag, Coni control RNAi, KO knockout, WT wild-type.

Considering that G3BP1 enhances RIG-I-mediated signaling, we hypothesized that G3BP1 affects the expression of RIG-I. We found that G3BP1 overexpression facilitated the expression of RIG-I but not IRF3 (Fig. 7a–b). To further investigate how G3BP1 upregulates RIG-I expression, we assessed the influence of 3-MA (inhibitor of the autophagy-dependent degradation pathway), NH_4_Cl (inhibitor of the lysosome-dependent degradation pathway), and MG132 (inhibitor of the ubiquitin-proteasome-dependent degradation pathway) on G3BP1-mediated upregulation of RIG-I in a mammalian overexpression system. The results indicated that MG132 inhibited G3BP1-mediated upregulation of RIG-I (Fig. 7c) and G3BP1 stabilized RIG-I via the ubiquitin-proteasome pathway.

It is widely accepted that E3 ubiquitin ligase enzyme RNF125 drives proteasomal degradation of RIG-I and negatively regulates innate antiviral immunity^13^. We next investigated whether G3BP1 is involved in the ubiquitination of RIG-I by RNF125. Ubiquitination assay results showed that G3BP1 overexpression diminished RNF125-mediated ubiquitination of RIG-I, whereas this function mediated by inactive mutant RNF125 (C72/75 A) was not affected by G3BP1 overexpression (Fig. 7d–e). In contrast, G3BP1-knockdown enhanced RIG-I ubiquitination (Fig. 7f). Moreover, we observed that G3BP1 overexpression inhibited K48-linked poly-ubiquitination of RIG-1 mediated by RNF125 (Fig. 7g). Furthermore, both knockdown and knockout of G3BP1 potentiated K48-linked poly-ubiquitination of RIG-1 in SeV-infected HEK293T cells (Fig. 7h–i). In addition, RNA pull-down experiment results indicated that G3BP1 overexpression could bind to 5´ppp-dsRNA and enhanced the binding of RIG-I to 5´ppp-dsRNA in vivo (Fig. 7j). These data suggest that G3BP1 stabilizes RIG-I by downregulating K48-linked ubiquitination of RIG-I mediated by RNF125 and enhances the binding of RIG-I to 5´ppp-dsRNA in vivo.

### G3BP1 promotes degradation of RNF125 via its auto-ubiquitination

**Fig. 8:**
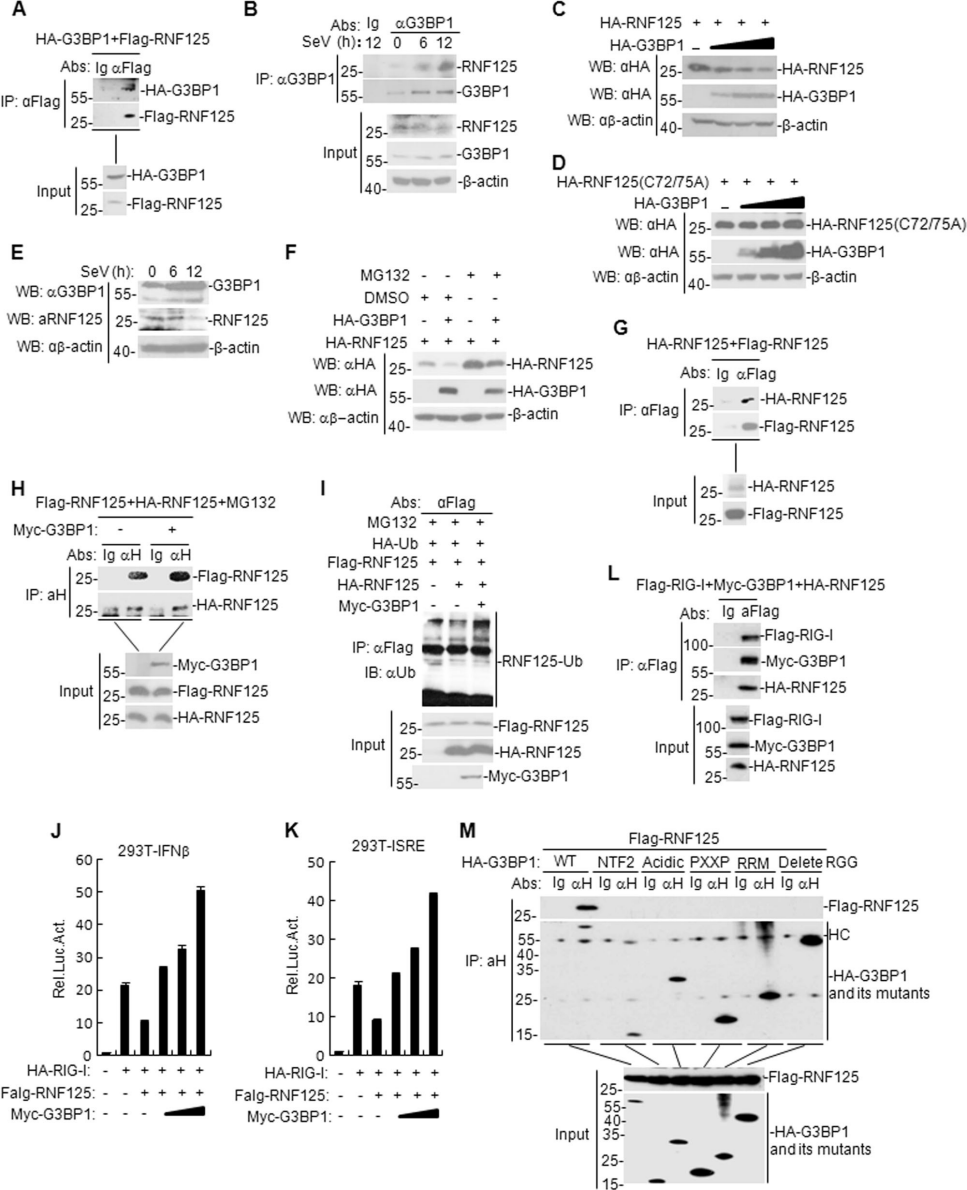
G3BP1 promotes degradation of RNF125 via its auto-ubiquitination. **a** Interaction between G3BP1 and RNF125 in the mammalian overexpression system. HEK293T cells were transfected with the indicated plasmids (5 μg of each) for 24 h. Co-IP and immunoblotting were performed with the indicated antibodies. **b** Endogenous G3BP1 interacted with RNF125 in HEK293T cells. HEK293T cells (5 × 10^7^) were untreated or infected with SeV for the indicated time. Co-IP and immunoblotting experiments were performed with the indicated antibodies. **c**, **d** Effects of G3BP1 on the expression of RNF125 or RNF125 (C72/75 A) mutant were evaluated. HEK293T cells were transfected with HA-G3BP1 (0, 0.5, 1.5, and 3 μg) and HA-RNF125 or HA-RNF125 (C72/75 A) plasmids (2 μg) for 24 h. Then the cell lysates were analyzed by immunoblotting with the indicated antibodies. **e** Effects of SeV infection on the expression of endogenous G3BP1 and RNF125 in HEK293T cells. HEK293T cells were uninfected or infected with SeV for the indicated time. The cell lysates were analyzed by immunoblotting with the indicated antibodies. **f** Effects of MG132 on G3BP1-mediated destabilization of RNF125. HEK293T cells (4 × 10^5^) were transfected with the indicated plasmids for 18 h and then the cells were treated with DMSO or MG132 for 6 h before immunoblotting analysis. **g** Interaction between RNF125 and RNF125 in the mammalian overexpression system. HEK293T cells were transfected with the indicated plasmids (5 μg of each). Co-IP and immunoblotting were performed with the indicated antibodies. **h** Effects of G3BP1 on the interaction between RNF125 and RNF125. The experiments were similarly to those described in **g**. **i** Effects of G3BP1 on the ubiquitination of RNF125. HEK293T cells (2 × 10^6^) were transfected with the indicated plasmids for 18 h and then treated with MG132 for 6 h. Co-IP and immunoblotting were performed with the indicated antibodies. **j**, **k** Effects of G3BP1 overexpression on RNF125-mediated RIG-I activation were assessed. HEK293T cells (1 × 10^5^) were transfected with the IFN-β reporter, ISRE (0.1 μg), HA-RIG-1 (100 ng), Flag-RNF125 (100 ng) or G3BP1 (0, 100, 200, and 400 ng) expression plasmids for 24 h before luciferase assays were performed. The experiment was repeated in triplicates. **l** Interaction between G3BP1, RIG-I, and RNF125 in HEK293T cells. HEK293T cells were transfected with the indicated plasmids for 24 h. Co-immunoprecipitation and immunoblotting analysis were performed with the indicated antibodies. **m** Interaction between G3BP1, G3BP1 mutants, and RNF125. The experiments were similarly to those described in **l**. Data are mean ± SD of three independent experiments. Co-IP Co-immunoprecipitation, EV, empty vector, Luc luciferase, αH anti-HA tag, HC heavy chain.

To confirm if there is an association between G3BP1 and RNF125, we first examined the interaction between G3BP1 and RNF125 in the mammalian overexpression system. Co-IP results showed that G3BP1 indeed interacted with RNF125 (Fig. 8a). This result was further corroborated by endogenous Co-IP experiments in SeV-infected HEK293T cells (Fig. 8b). Additionally, we found that G3BP1 suppressed RNF125 expression in a dose-dependent manner, whereas the expression of RNF125 mutant (C72/75 A) was not affected (Fig. 8c–d). Moreover, we found that the G3BP1 expression was enhanced following SeV stimulation in HEK293T cells, whereas RNF125 expression was reduced following SeV stimulation in HEK293T cells (Fig. 8e). We next found that MG132 inhibited RNF125 degradation in G3BP1-overexpressed HEK293T cells (Fig. 8f). To further investigate how G3BP1 degrades RNF125, we examined the oligomerization and auto-ubiquitination of RNF125 after G3BP1 transfection. The results showed that RNF125 formed a homo-dimer (Fig. 8g) and G3BP1 increased RNF125 homo-dimerization (Fig. 8h). Furthermore, we observed that G3BP1 promoted auto-ubiquitination of RNF125 (Fig. 8i). Further results showed that RNF125 inhibited RIG-I-triggered activation of the IFN-β promoter and ISRE, whereas G3BP1 was able to reverse this inhibition (Fig. 8j–k). In transient transfection and co-immunoprecipitation experiments, we found that RIG-I interacted with G3BP1 and RNF125 in HEK293T cells (Fig. 8l). In addition, we also found that G3BP1 but not G3BP1 NTF2, G3BP1 acidic domain, G3BP1 PXXP, G3BP1 RRM, and G3BP1 (delete RGG) interacted with RNF125 (Fig. 8m). Taken together, these results suggest that G3BP1 degrades RNF125 by promoting RNF125-mediated oligomerization and auto-ubiquitination.

## Discussion

The cytoplasmic viral RNA sensor RIG-I is a receptor protein, playing critical roles in the recognition of cytoplasmic dsRNA and activation of IRFs as well as NF-κB^34^. It is well known that RIG-I-deficient cells fail to recognize various RNA viruses, such as paramyxoviruses, influenza virus, and VSV^35,36^. Consistently, RIG-I knockout mice have been reported to be more susceptible to VSV, hepatitis C virus (HCV), and Japanese encephalitis virus^6^. The protein level of RIG-I is also delicately regulated during virus infection to ensure the optimal activation and timely termination of innate immune responses. However, the molecular mechanism of RIG-I activity regulated by post-translational modifications has not been fully elucidated. In this study, we found that G3BP1 acts as a positive regulator of RIG-I, dynamically maintaining the stability of RIG-I and ensuring that a proper innate immune response is mounted against RNA viruses.

Previously, it has been shown that G3BP1 overexpression contributes to the IFN-β production induced by RIG-I^25^. Consistent with this observation, we first found that G3BP1 overexpression potentiates SeV- and poly (I:C)-triggered induction of IFN-β, whereas knockdown or knockout of G3BP1 exhibits the opposite effect. We further identified that G3BP1 is able to interact with RIG-I and boost its expression. The RING-type E3 ubiquitin ligase RNF125, also known as T cell RING Activation protein 1 (TRAC-1), targets RIG-I for its subsequent proteasomal degradation^13,37,38^. Our data suggest that G3BP1 maintains RIG-I levels by antagonizing RNF125-mediated degradation of RIG-I. Firstly, RIG-I expression could be stabilized by G3BP1 via antagonizing RNF125-mediated RIG-I degradation. Secondly, we demonstrated that G3BP1 potentiates the self-association and auto-ubiquitination of RNF125. Hence, it is more likely that G3BP1 first promotes RNF125 degradation by enhancing self-association and auto-ubiquitination of RNF125, and then RIG-I degradation mediated by RNF125 is alleviated.

Previous studies have suggested that G3BP1 is involved in the life cycle of various RNA viruses. For example, G3BP1 directly interacts with foot-and-mouth disease virus IRES and negatively regulates IRES function^39^. The overexpression of G3BP1-SGs negatively regulates the replication of coxsackievirus type B3 at the RNA, protein, and viral progeny levels^40^. Besides, G3BP1 also inhibits mammalian orthoreovirus replication during viral infection^41^. Interestingly, a recent study has revealed that G3BP1 binds to viral dsRNA and RIG-I to enhance IFN-β response^25^. Consistently, we found that G3BP1 could bind to viral dsRNA and enhance the binding of RIG-I to dsRNA in vivo. Notably, our data reveal that G3BP1 could enhance RIG-I expression by inhibiting RIG-I ubiquitination. In particular, although Kim et al., demonstrate that G3BP1 directly binds RIG-I via the C-terminal RGG domain of G3BP1^25^, there is no evidence to show that G3BP1 could enhance RIG-I to bind dsRNA in vitro and it is likely that G3BP1 increases RIG-I expression to enhance the binding of RIG-I to dsRNA. Therefore, it will be important to test whether G3BP1 is a co-sensor to promote RIG-I recognition of pathogenic RNA in vitro, in the future. Taken together, upon infection with RNA viruses, G3BP1 increases ubiquitination of RNF125 to attenuate RNF125 expression, thereby promoting the expression of RIG-I. These results provide important insights into the molecular mechanisms of innate immune responses mounted against RNA viruses.

## Supplementary information

Supplementary figures legends

Supplementary figure 1

Supplementary figure 2

Supplementary figure 3

Supplementary figure 4
