# Supplementary material for: G3BP1 inhibits RNA virus replication by positively regulating RIG-I-mediated cellular antiviral response
Source: Cell Death Dis. 2019 Dec 11;10(12):946. doi: 10.1038/s41419-019-2178-9 (PMC6906297; doi:10.1038/s41419-019-2178-9)
Supplement: Supplementary file 1 — Supplementary figures legends [file 41419_2019_2178_MOESM1_ESM.doc]

**Supplemental Fig. 1** **Effects of G3BP1 overexpression on the transcription of antiviral genes, related to Fig. 1.** **a–g** Effects of G3BP1 overexpression on SeV-induced transcription of downstream genes. HEK293T cells stably overexpression G3BP1 were infected with SeV for 12 h before real-time PCR analysis was perferomed. **h–n** Effects of G3BP1 overexpression on cytoplasmic poly (I:C)-induced transcription of downstream genes. HEK293T cells stably overexpressing G3BP1 were transfected with poly (I:C) (1 μg/ml) for 18 h before real-time PCR analysis was performed. The experiment was repeated in triplicates. Data are mean ± SD, n=3. *p<0.05, **p<0.01, two-tailed t-test. Con, control; Rel., relative.

**Supplemental Fig. 2** **The effects of G3BP1 knockdown on the transcription of antiviral genes, related to Fig. 2.** **a–n** Effects of G3BP1-RNAi on SeV- or poly (I:C)-induced transcription of downstream genes. Stable G3BP1-knockdown HEK293T cells were infected with SeV **a–g** or transfected with poly (I:C) **h–n** (1 μg/ml) for 12 h or 18 h before real-time PCR analysis was performed. The experiment was repeated in triplicates. Data are mean ± SD, n=3. *p<0.05, **p<0.01, two-tailed t-test. Coni, control-RNAi; Rel., relative.

**Supplemental Fig. 3** **Effects of G3BP1 deficiency on virus-induced transcription of downstream genes, related to Fig. 3.** **a–g** G3BP1-deficient HEK293T cells were infected with SeV for the indicated time before qPCR analysis was performed. **h–n** Effects of G3BP1 deficiency on poly (I:C)-induced transcription of downstream genes. G3BP1-deficient HEK293T cells were transfected with poly (I:C) for 18 h before qPCR analysis was performed. The experiment was repeated in triplicates. Data are mean ± SD, n=3. *p<0.05, **p<0.01, two-tailed t-test. WT, wild type; Rel., relative.

**Supplemental Fig. 4** **qPCR analysis of RLR-mediated induction of downstream antiviral genes in G3BP1-deficient HEK293T cells reconstituted with G3BP1, related to Fig. 3.** **a–n** The G3BP1-deficient HEK293T cells reconstituted with G3BP1 or empty vector were left uninfected or infected with SeV for 12 h or were transfected with poly (I:C) for 18 h before qPCR experiments were performed. Expression of the transduced proteins was detected by immunoblotting with the indicated Abs. The experiment was repeated in triplicates. Data are mean ± SD, n=3. *p<0.05, **p<0.01, two-tailed t-test. WT, wild type; Rel., relative.
