# Supplementary figures and images for: G3BP1 inhibits RNA virus replication by positively regulating RIG-I-mediated cellular antiviral response
Source: Cell Death Dis. 2019 Dec 11;10(12):946. doi: 10.1038/s41419-019-2178-9 (PMC6906297; doi:10.1038/s41419-019-2178-9)

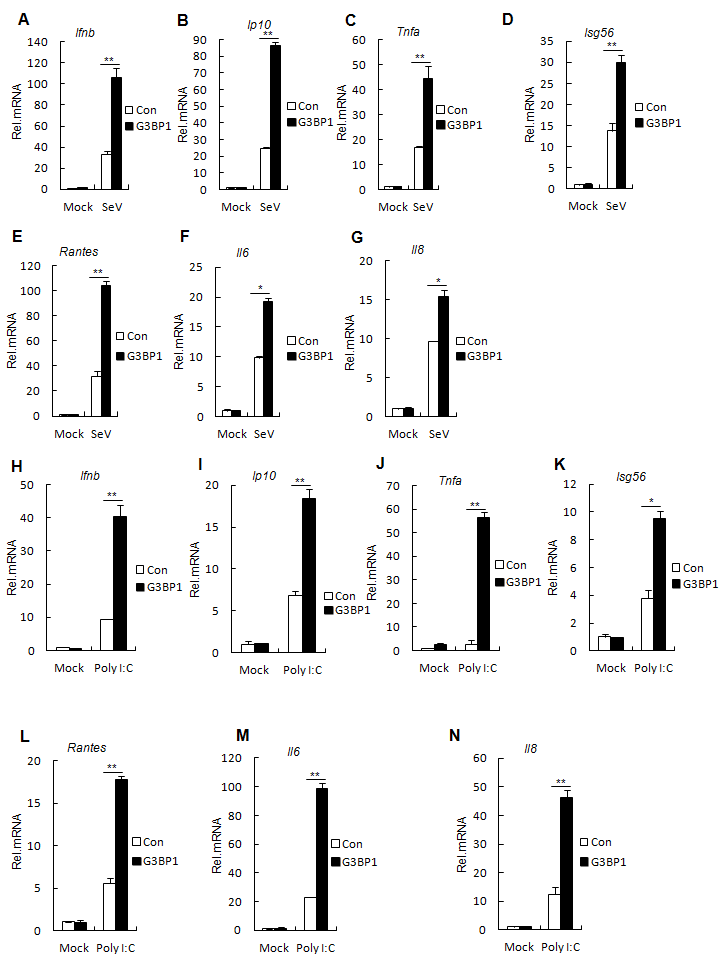

Supplement: Supplementary file 2 — Supplementary figure 1 [file 41419_2019_2178_MOESM2_ESM.tif]

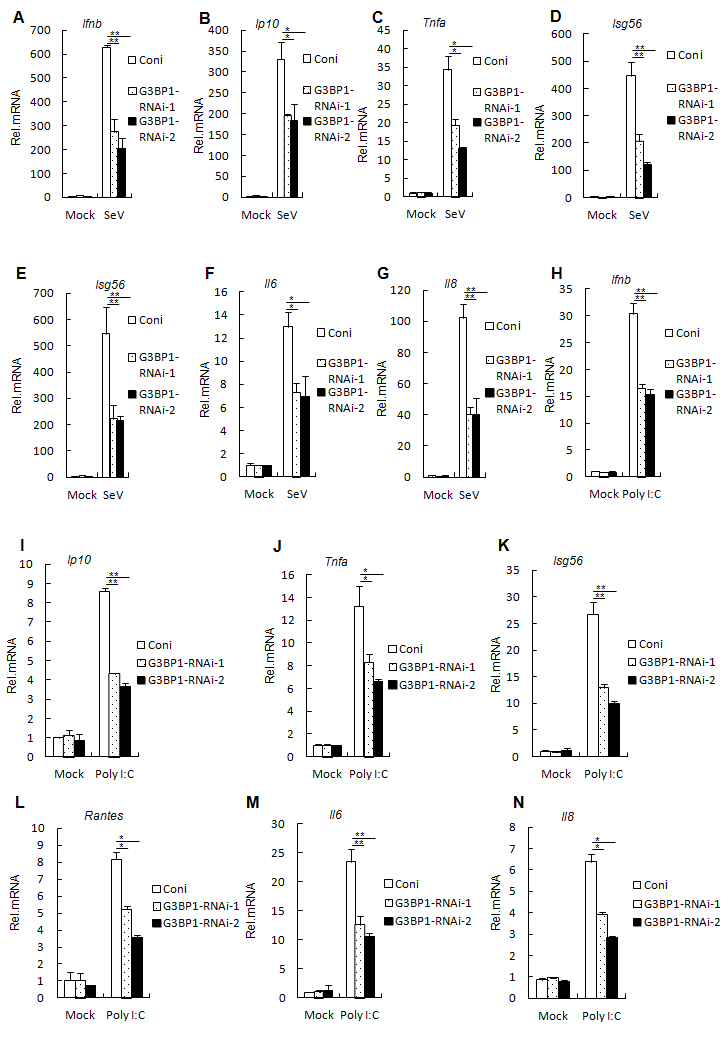

Supplement: Supplementary file 3 — Supplementary figure 2 [file 41419_2019_2178_MOESM3_ESM.tif]

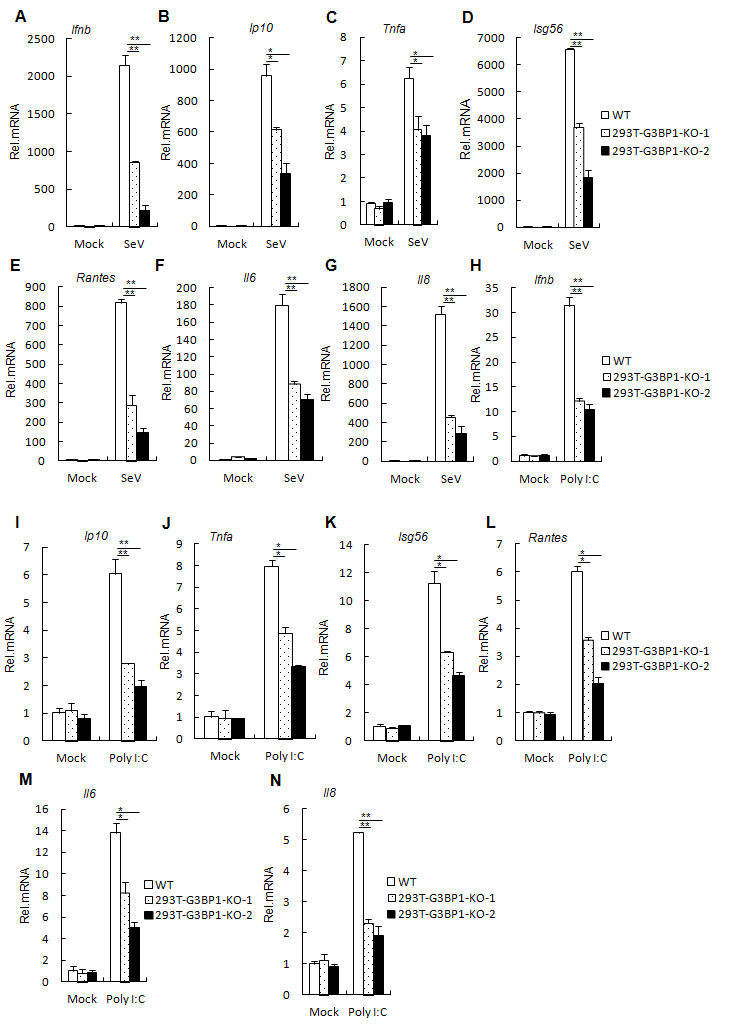

Supplement: Supplementary file 4 — Supplementary figure 3 [file 41419_2019_2178_MOESM4_ESM.tif]

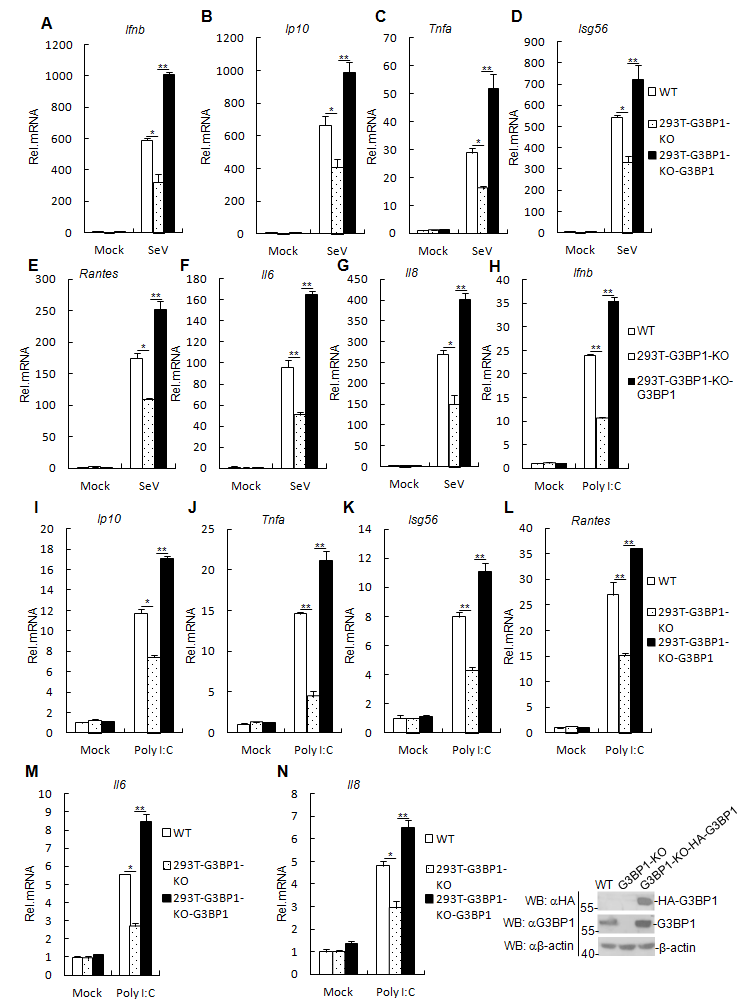

Supplement: Supplementary file 5 — Supplementary figure 4 [file 41419_2019_2178_MOESM5_ESM.tif]
